# Patrolling monocytes mediate virus neutralizing IgG effector functions: beyond neutralization capacity

**DOI:** 10.3389/fimmu.2025.1600056

**Published:** 2025-05-29

**Authors:** Abdelrahman Elwy, Swati Dhiman, Hossam Abdelrahman, Julia Specht, Theresa Charlotte Christ, Julia Falkenstein, Harpreet Kaur, Lisa Holnsteiner, Judith Lang, Matthias Mack, Falk Nimmerjahn, Wiebke Hansen, Karl Sebastian Lang

**Affiliations:** ^1^ Institute of Immunology, Medical Faculty, University of Duisburg-Essen, Essen, Germany; ^2^ Institute of Medical Microbiology, University Hospital Essen, University Duisburg-Essen, Essen, Germany; ^3^ Department of Nephrology, University Hospital Regensburg, Regensburg, Germany; ^4^ Division of Genetics, Department of Biology, University of Erlangen-Nürnberg, Erlangen, Germany

**Keywords:** IgG, neutralizing antibodies, patrolling monocytes, LCMV, passive immunization

## Abstract

Neutralizing antibodies (nAbs) are pivotal in developing fast, broadly protective therapeutics against novel pandemic viruses. Despite their well-known direct neutralization capacity, their effector mechanisms via Fc receptors remain poorly understood. Identifying the types of effector cells engaged in antibody-mediated effector functions is essential for regulating their activities. Using the lymphocytic choriomeningitis virus (LCMV), we show that nAbs obtained from immune sera or monoclonal LCMV-specific nAbs show dependency on Fc receptors. We demonstrate that therapy with nAbs is highly protective in the presence of patrolling monocytes. These monocytes bind nAbs primarily via FcγRIV, targeting virus-infected cells, and thereby limiting virus propagation. Depleting patrolling monocytes or blocking FcγRIV resulted in a substantial loss of virus control by nAbs, indicating the pivotal role of patrolling monocytes in the antiviral activity of these antibodies. In conclusion, our findings highlight that, alongside direct neutralization, nAbs primarily exert their effects through the involvement of patrolling monocytes.

## Introduction

Viral infections pose significant challenges to human health, necessitating robust immune responses for control and eradication. The absence of vaccines for several significant viral diseases underscores the urgency for developing therapeutics to preserve lives and manage epidemics. While active immunization remains a cornerstone in preventive medicine, the prolonged timeline for vaccine development, and the emergence of novel viral strains presents formidable hurdles. In such scenarios, passive immunization via antibody administration offers immediate protection and proves particularly advantageous in situations demanding rapid intervention, such as outbreaks or instances where individuals cannot mount an effective immune response independently.

Antibodies, particularly those belonging to the IgG subclass, are pivotal in combating viruses by targeting and neutralizing them (1). Numerous mechanisms contribute to the protective functions of IgG antibodies (2–5). Some functions, like neutralization, are independent of the fragment crystallizable domain (Fc). Others, such as antibody-dependent cellular phagocytosis (ADCP), and antibody-dependent cell-mediated cytotoxicity (ADCC), rely on interactions between the Fc domain of the antibody and other proteins (such as complement system) or immune effector cells through Fc receptor recognition. Indeed, the interaction between the Fc portion of antibodies and effector cells expressing Fcγ receptors ( FcγRs) during viral infection remains a field of ongoing investigation.

In mice, the family of FcγRs comprises three activating (FcγRI, FcγRIII, FcγRIV) and one inhibitory (FcγRIIB) member (6). Similarly, non-human primates and humans possess orthologous proteins with analogous functions. Numerous studies have aimed to elucidate the involvement of activating FcγRs in mediating the activity of various IgG subclasses. From these investigations, a discernible pattern has emerged: while the activity of IgG1 relies solely on activating FcγRIII, the effector functions induced by the potent IgG subclasses IgG2a or c and IgG2b appear to be predominantly dependent on FcγRIV. Alternatively, combinations of FcγRI and FcγRIV, or FcγRIII and FcγRIV may also contribute to these effector functions (7–12).

Studies have demonstrated that FcγRs play a central role in enhancing the antiviral activity of IgG antibodies by facilitating processes such as ADCC, ADCP, and complement-dependent cytotoxicity (CDC) (13, 14). Through their interaction with FcγRs, antibodies can engage effector cells such as natural killer (NK) cells, macrophages, and neutrophils, leading to the elimination of virus-infected cells and control of viral dissemination within the host (15–18). Moreover, emerging evidence implicates monocytes, a subset of innate immune cells, in the regulation of antiviral immunity during viral infections (19). Nevertheless, their specific role in combating viruses during passive immunization remains unclear. Given their wide expression pattern of FcγRs, particularly their high expression of FcγRIV (20), investigating their role in IgG-mediated virus control is of utmost importance.

Patrolling monocytes play a crucial role in surveilling capillaries and removing micrometric particles from the luminal side in steady-state conditions (21). These cells exhibit high expression levels of FcγRIV compared to other cell types in the blood, while neutrophils demonstrate comparatively lower expression of FcγRIV. Indeed, an elegant study by Biburger et al., 2011 (20), has emphasized the essential role of patrolling monocytes in orchestrating IgG-mediated platelet and B-cell depletion *in vivo* under sterile conditions. Nevertheless, their role in orchestrating IgG-mediated virus clearance is yet to be explored.

The role of non-neutralizing antibodies in clearing infections has been established (22–24) along with the identification of the effector cells involved (25). Conversely, the effector cells involved in Fc-mediated control of infection by nAbs remain uninvestigated. This gap in research is largely attributed to the assumption that viruses are neutralized by nAbs, overlooking the potential role of Fc receptors in mediating antibody-dependent effector functions.

Various studies are exploring the role of nAbs *in vivo* (26–29), while it was mainly considered that the antiviral protection is achieved by direct neutralization of the virus, the role of Fc-mediated processes might be underestimated. With the diverse functions of Fc receptors, we consider that the identification of cell populations participating in IgG-dependent virus control remains an important area for further investigation.

In this study, we aim to investigate the intricate mechanisms underlying nAb-mediated control of viral infections, with a particular focus on the effector cells involved. Our model system utilizes Lymphocytic Choriomeningitis Virus (LCMV). Through targeted depletion and the utilization of various mouse models, we have elucidated the critical role of patrolling monocytes in the effective control of LCMV via virus-specific antibodies. These findings hold significant implications for the development of innovative therapeutic approaches aimed at enhancing antiviral immunity and attenuating the detrimental effects of acute viral infections.

## Results

### Passive nAbs treatment relies on activatory FcγRs

**Figure 1:**
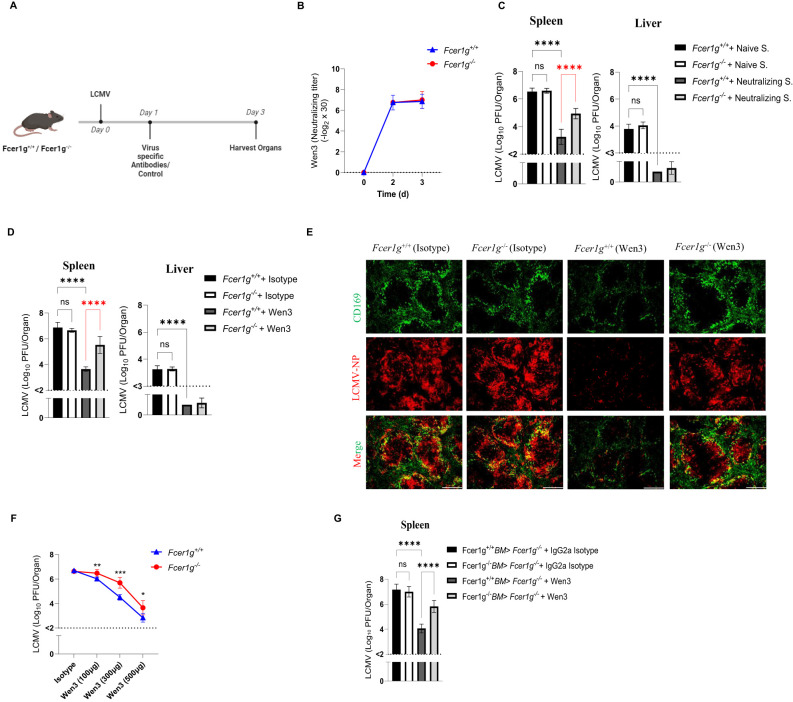
Passive neutralizing antibodies treatment relies on activatory FcγRs. **(A)** Schematic presentation of the experiments in **(B-E)**. Fcer1g^+/+^ (WT) and Fcer1g^-/-^ (FcγR-KO) mice were infected intravenously on day 0 with 2 × 10^5^ PFU (plaque forming units) of LCMV-WE. On day 1 post-infection, mice were treated with virus-specific antibodies (either polyclonal serum from LCMV-immune mice or the monoclonal neutralizing antibody Wen3) or respective control antibodies. Mice were euthanized 3 days post infection (d.p.i.), and spleen and liver were collected for further analyses. **(B)** Neutralizing capacity of Wen3 (3 d.p.i.) from serum of WT (Fcer1g^+/+^) and FcγR-KO (Fcer1g^-/-^) mice infected with LCMV-WE (2x10^5^ PFU) on day 0 and treated on day 1 with Wen3 (350 µg). Results show pooled data from two independent experiments with similar results (n=3–4 mice/group/experiment). Titres are presented as twofold dilution steps (log2) times the predilution (x 30). **(C, D)** Virus titres (3 d.p.i.) in spleen and liver of WT (Fcer1g^+/+^) and FcγR-KO (Fcer1g^-/-^) mice infected with LCMV (2x10^5^ PFU) on day 0 and treated on day 1 with **(C)** neutralizing serum (Neutralizing S.) or naïve serum (Naïve S.) (see methods), **(D)** Wen3 (350 µg) or isotype (IgG2a). Results show the pooled data from three independent experiments with similar results. (n=2–4 mice/group/experiment). **(E)** Spleen sections collected 3 d.p.i. were stained for CD169 (green) and LCMV nucleoprotein (−NP) (red). WT (Fcer1g^+/+^) and FcγR-KO (Fcer1g^-/-^) mice infected with LCMV (2x10^5^ PFU) on day 0 and treated on day 1 with Wen3 (350 µg) or isotype (IgG2a). Shown are representative pictures from three independent experiments (n=2–4 mice/group/experiment). Scale bar= 300 µm. **(F)** Virus titres (3 d.p.i.) in spleen of WT (Fcer1g^+/+^) and FcγR-KO (Fcer1g^-/-^) mice infected with LCMV (1x10^5^ PFU) on day 0 and treated on day 1 with different concentrations of Wen3 (100, 300, 500 µg) or isotype (IgG2a) (500 µg). Results show the data from one experiment, experiment was repeated twice with similar results. (n=4–5 mice/group/experiment). **(G)** Virus titres (3 d.p.i) in spleen of FcγR-KO mice transplanted with Fcer1g^+/+^ bone marrow (BM) or Fcer1g^-/-^ BM. Mice were infected with LCMV-WE (2x10^5^ PFU) on day 0 and treated on day 1 with Wen3 (350 µg) or isotype (IgG2a). Results show the pooled data from two independent experiments with similar results. (n=3–4 mice/group/experiment). Statistical analysis was performed by using the One-way ANOVA test **(C, D, G)** or Student’s t-test **(F)**. *p < 0.05, **p < 0.01, ***p < 0.001, ****p < 0.001. Horizontal dotted lines indicating the detection limit. ns, non-significant.

The influence of activatory FcγRs on virus nAbs remains relatively understudied, particularly due to the assumption that viruses are directly neutralized via Fab-dependent mechanisms, overlooking the potential role of activatory FcγR during viral clearance. Given the decisive role of activatory FcγRs in mediating IgG-dependent effector mechanisms, we aimed to evaluate their impact on virus nAbs during an acute viral infection. Initially, we confirmed the neutralizing efficacy of two types of antibodies: broad nAbs obtained from sera of immunized mice, and a previously described monoclonal LCMV nAb, Wen3 (**Supplementary Figure S1A**) (29). Our assessment affirmed robust neutralizing activity for both antibody types. First, to rule out variations in antibody distribution or degradation between WT and Fcer1g knockout mice (*Fcer1g^-/-^*, commonly known as FcγR-KO) lacking all activatory FcγRs, we isolated serum from actively immunized mice and evaluated its neutralizing activity. Remarkably, we observed similar neutralizing capacities in the serum from both passively immunized WT and Fcer1g-KO mice (**Figures 1A, B**). By excluding variations in antibody concentration and distribution, we can more confidently attribute any observed differences in virus control to the absence of activatory FcγR rather than fluctuations in antibody levels.

Subsequently, by utilizing WT and FcγR-KO mice, our results unveiled a significant dependence on activatory FcγRs for early virus titre control in the spleen but not in the liver by both broad nAbs (obtained from LCMV immune mice) (**Figure 1C**), and Wen3 (**Figures 1D, E**). Since the liver titre was undetectable in these experiments, we refrained from measuring it in future experiments. Interestingly, we observed a more pronounced reduction in virus titres in the spleen of WT mice compared to FcγR-KO mice. This suggests a dual mechanism of action for virus nAbs, with the direct neutralization of the virus accounting for the decrease observed in KO mice, and an additional Fc-mediated effect contributing to the further reduction seen in WT mice. These findings underscore the dependency on FcγRs specifically in the spleen, emphasizing the crucial role of FcγR in mediating the efficacy of virus nAbs. In line with these findings, levels of interferon-γ (IFNγ) and interferon-α (IFNα) were notably reduced in WT mice treated with Wen3 compared to FcγR-KO mice (**Supplementary Figures S1B**).

To further explore the dose-dependent effect of Wen3 on virus titre reduction, we administered three different doses (100 µg, 350 µg, and 500 µg) of Wen3 to both WT and FcγR-KO mice, strikingly, at the lowest concentration, the FcγR-KO mice did not exhibit any reduction in virus titres, suggesting that at lower concentrations, nAbs primarily exert their function through FcγRs (**Figure 1F**). Conversely, at medium doses, both direct neutralization and Fc-mediated mechanisms appear to contribute to virus titre reduction.

Next, we aimed to investigate whether nAbs depend on activatory FcγRs when administered before infection. Our findings revealed significant differences in virus control between WT and KO groups, even in the presence of pre-treatment with memory serum or Wen3 (**Supplementary Figures S1C, D**). Collectively, these results underscore the inadequacy of nAbs alone in conferring effective virus control and emphasize the critical contribution of activatory FcγRs-mediated mechanisms.

To further pinpoint the effect of activatory FcγRs on hematopoietic cells, we generated bone marrow chimeras by irradiating FcγR-KO mice followed by reconstitution with either WT or FcγR-KO bone marrow cells. Notably, chimeric mice receiving WT bone marrow exhibited a significant decrease in virus levels following Wen3 treatment compared to those receiving KO bone marrow (**Figure 1G**), providing additional evidence for the pivotal role of activatory FcγRs on hematopoietic cells in controlling virus infection through virus neutralizing IgG antibodies.

### Passively transferred nAbs bind exclusively to monocyte subsets

**Figure 2:**
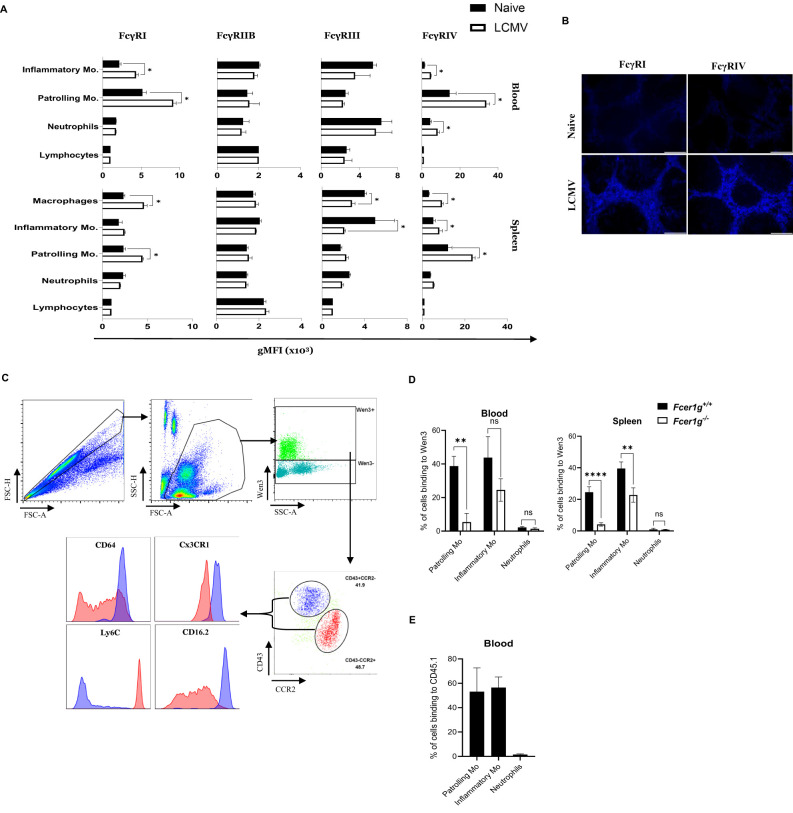
Passively transferred nAbs bind exclusively to monocyte subsets **(A)** Graphs represent the geometric mean fluorescence intensity (gMFI) of the indicated FcγR (FcγRI, FcγRIIB, FcγRIII, and FcγRIV) on lymphocytes, neutrophils, patrolling monocytes, and inflammatory monocytes in blood as well as macrophages in the spleen from naive and LCMV infected mice (2x10^5^ PFU) (24 hours post infection (h.p.i.)). See (**Supplementary Figure S2**) for gating strategy of the monocyte subsets. Results show the data from one experiment, experiment was repeated twice with similar results. (n=4 mice/group/experiment). **(B)** Spleen sections collected from naïve and LCMV infected mice (2x10^5^ PFU) (24 h.p.i.) were stained for FcγRI and FcγRIV. Shown are representative pictures from three independent experiments (n=4 mice/group/experiment). **(C)** Flow cytometry plots depict the binding of fluorescently labeled LCMV-specific antibody Wen3 to distinct cell populations in blood, captured 25 minutes after injection into LCMV-infected mice. Two distinct cell populations; Inflammatory and patrolling monocytes were identified based on their expression of CD43 and CCR2, denoted as CD43^+^CCR2^-^ (Blue) and CD43^-^CCR2^+^ (Red). Arrows extend from these two populations to indicate their respective expression levels for Ly6C, Cx3CR1, CD64 (FcγRI), and CD16.2 (FcγRIV) on histograms. Shown are representative plots from three independent experiments (n=4 mice/group/experiment). **(D)** Binding percentage of Wen3-labeled (FITC) antibody to patrolling monocytes(-Mo), inflammatory monocytes(-Mo), and neutrophils 25 minutes after intravenous injection. WT (Fcer1g^+/+^) and FcγR-KO (Fcer1g^-/-^) mice were infected with LCMV-WE (2x10^5^ PFU) for 24 hours before injection of labeled Wen3. Results show the data from one experiment, experiment was repeated three times with similar results. (n=4 mice/group/experiment). **(E)** Graph represents the binding percentage of the fluorescently labeled CD45.1 (IgG2a) antibody to the indicated cell populations in the blood 25 min after injection to LCMV infected WT mice. Results show the data from one experiment, experiment was repeated twice with similar results. (n=4 mice/group/experiment). Statistical analysis was performed by using Student’s t-test **(A, D)**. *p < 0.05, **p < 0.01, ****p < 0.001, ns, non-significant.

Next, we aimed to capture the overall changes in FcγRs expression levels following viral challenge. Hence, the FcγR expression repertoire on effector cells following infection might provide insights into responsible effector cell populations. To explore this, we assessed the expression of all FcγRs on different immune cells in naïve state as well as 24 hours after infection in both blood and spleen (**Figures 2A, B**). The gating strategy utilized to gate different monocyte subsets is detailed in **Supplementary Figures S2A, B**. Interestingly, we observed a significant increase in the expression of FcγRI and FcγRIV on patrolling monocytes in both spleen and blood (**Figure 2A, B**), as well as on splenic macrophages, 24 hours post-infection compared to their naïve counterparts. This finding suggests a plausible involvement of these receptors in facilitating antibody-dependent effector functions crucial for viral clearance.

IgG2a antibodies, such as Wen3, are known for their potent activation of effector functions through specific FcγR binding, mainly FcγRIV (30, 31). To explore this, we administered FITC-labeled Wen3 antibody to mice infected with LCMV. Strikingly, our analysis revealed predominant binding of Wen3 to two distinct populations: patrolling and inflammatory monocytes (**Figure 2C**). Building on these findings, our investigation aimed to ascertain whether Wen3 binds to monocyte subsets via FcγRs. To investigate this, we administered FITC-labeled Wen3 to both FcγR WT and KO mice. Subsequent analysis, conducted 25 minutes post-injection, demonstrated significant binding of Wen3 to both patrolling and inflammatory monocyte populations in both blood and spleen (**Figure 2D**). Notably, the binding of Wen3 to patrolling monocytes was almost exclusively dependent on FcγR, whilst binding to inflammatory monocytes did only partially depend on activatory FcγRs (**Figure 2D**). Moreover, minimal binding to neutrophils was observed indicating a selective interaction with monocyte subsets.

To determine the role of the variable region of Wen3 in influencing the binding to monocyte subsets, we injected FITC-labeled IgG2a anti-CD45.1 antibody into LCMV infected CD45.2 mice. Remarkably, we observed a similar binding pattern (**Figure 2E**), confirming that the interaction is mediated via the Fc region. These results strongly suggest that activatory FcγRs on monocyte subsets might play a role in controlling virus infection through nAbs.

### Macrophages and inflammatory monocytes are dispensable for nAbs-mediated LCMV control

**Figure 3:**
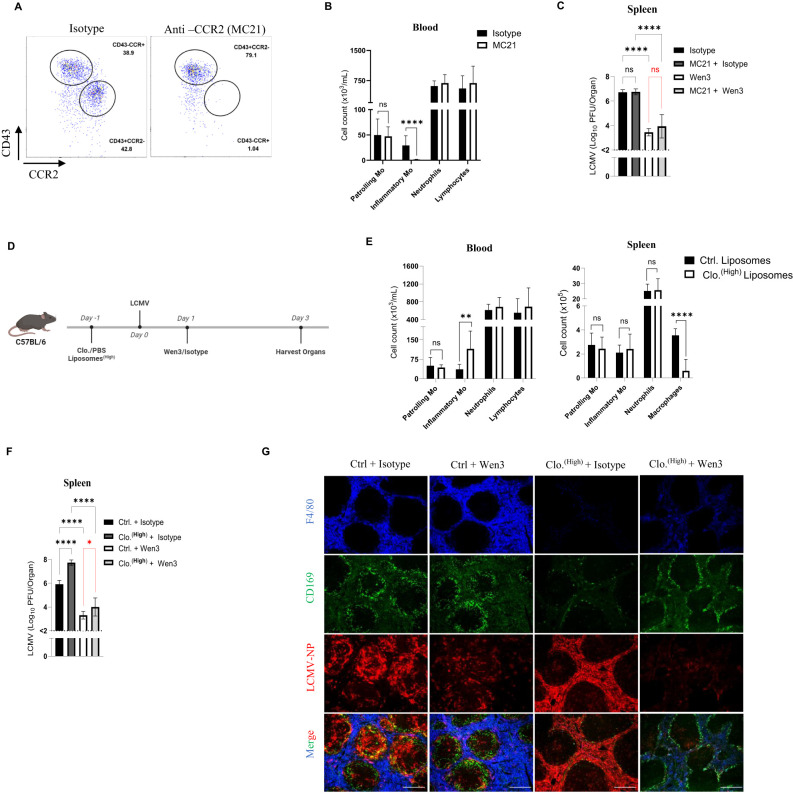
Macrophages and inflammatory monocytes are dispensable for nAbs-mediated LCMV control. **(A)** Flow cytometric analysis of monocytes subsets, CD45^+^Lin^-^(CD45R^+^ CD8^+^CD4^+^NK^+^), CD11b^+^Ly6G^-^, in the peripheral blood of MC21 or isotype (rat IgG2a)-treated mice 3 days pot infection (d.p.i). Mice were infected with LCMV-WE (2x10^5^ PFU) on day 0, preceded by treatment with 25 µg MC21 or isotype (rat IgG2b) on day -1 and again on day 1. See (**Supplementary Figure S2**) for gating strategy of the monocyte subsets. **(B)** Numbers of patrolling monocytes (-Mo), inflammatory monocytes (-Mo), neutrophils, and lymphocytes in the peripheral blood of MC21 or isotype (rat IgG2a)-treated mice 3 d.p.i. Results show the pooled data from two independent experiments with similar results. (n=3–5 mice/group/experiment). **(C)** Virus titres (3 d.p.i) in spleen of WT mice infected with LCMV-WE (2x10^5^ PFU) on day 0 and treated on day 1 with Wen3 (350 µg) or isotype (IgG2a), On day -1 and day 1 mice were treated with 25 µg MC21 or isotype (rat IgG2b). Results show the pooled data from two independent experiments with similar results. (n=3–5 mice/group/experiment). **(D)** Schematic presentation of the experiments in **(E-G)**. **(E)** Numbers of patrolling monocytes (-Mo), inflammatory monocytes (-Mo), neutrophils, and lymphocytes in the peripheral blood and spleen (3 d.p.i.) of mice treated with 10 µL/g bodyweight control or clodronate liposomes one day before LCMV infection (2x10^5^ PFU). Results show the pooled data from two independent experiments with similar results. Experiment was repeated three times with similar results (n=4–5 mice/group/experiment). **(F)** Shown are the virus titres (3 d.p.i.) in spleen of WT mice infected with LCMV-WE (2x10^5^ PFU) on day 0 and treated on day 1 with Wen3 (350 µg) or isotype (IgG2a). On day -1 mice were treated with 10µL/g bodyweight control or clodronate liposomes. Results show the pooled data from two independent experiments with similar results. Experiment was repeated three time with similar results (n=3–5 mice/group/experiment). **(G)** Spleen sections collected 3 d.p.i. were stained for F4/80 (blue), CD169 (green), and LCMV nucleoprotein (−NP) (red). Scale bar 300 µm. Shown are representative pictures from three independent experiments (n=2–4 mice/group/experiment). Statistical analysis was performed by using the One-way ANOVA test **(C, F)** or Student’s t-test **(B, E)**. *p < 0.05, **p < 0.01, ***p < 0.001, ****p < 0.001, ns, non-significant. Horizontal dotted lines indicating the detection limit.

Next, we aimed to determine which effector cells, as well as activatory receptors, are responsible for Fc-mediated effector functions. Our earlier observations indicate that the LCMV nAb Wen3 binds to both patrolling and inflammatory monocytes via Fc receptors. To determine whether inflammatory monocytes play a role in mediating the function of Wen3, we selectively depleted inflammatory monocytes using a well-established antibody targeting CCR2 (MC-21), known for effectively removing inflammatory monocytes while leaving patrolling monocytes unaffected (32, 33). Successful depletion of inflammatory monocyte was confirmed in blood (**Figures 3A, B**); while patrolling monocytes population remained intact (**Figure 3B**). Interestingly, depletion of inflammatory monocytes did not hinder Wen3-mediated virus control (**Figure 3C**), reinforcing the dispensability of inflammatory monocytes in this context.

Given the crucial role of macrophages in phagocytosis of opsonized virus particles as well as their ability to express all classes of FcγRs (34), we sought to investigate the contribution of macrophages in nAb-mediated virus control during LCMV infection. To specifically evaluate the effect of macrophages, we employed a previously described method capitalizing on the rapid regeneration of monocytes in the blood (35). Macrophages were depleted using a single dose of clodronate (**Figure 3D**), ensuring the depletion of tissue-resident macrophages throughout the infection period while facilitating earlier monocyte appearance (**Figure 3E**). Indeed, macrophage depletion led to increased LCMV propagation (**Figures 3F**), consistent with the known protective role of type 1 interferon production by macrophages (36). Nevertheless, and strikingly, even in the absence of tissue-resident macrophages and the heightened virus propagation, in presence of monocytes subsets, passive administration of Wen3 provided comparable protection to control-treated mice (**Figures 3F, G**). This underscores the dispensability of inflammatory monocytes as well as the macrophages in antibody-mediated virus control. Moreover, it highlights the multifaceted nature of the immune response, prompting further investigation into the specific effector mechanisms responsible for antibody-mediated viral clearance.

### Neutrophils, NK cells and T-cells are dispensable for nAbs-mediated virus control

Next, we aimed to explore the involvement of other innate and adaptive immune cells in Fc-mediated control of LCMV through Wen3. Neutrophils were initially considered due to their previously described role in controlling viruses via FcγRs (37). Additionally, they express FcγRIV, which is crucial for IgG2a activity (11), making them ideal candidates for effector function. Interestingly, depletion of neutrophils did not affect the effectiveness of Wen3 (**Supplementary Figure S3A**), suggesting that neutrophils are dispensable for FcγR-mediated virus control. Moreover, we assessed the roles of NK cells and T-cells, two key components of the immune system known for their cytotoxic functions and ADCC (38). These cell types were included not only due to their well-established effector functions but also to explore the broader immunological landscape potentially engaged by monoclonal antibody therapy. NK cells, through FcγRIII, can directly mediate ADCC (39), while T cells may be indirectly activated via cytokines produced by other immune cells following mAb engagement, contributing to immune orchestration and viral clearance (40). Utilizing NK cell-depleted and T-cell knockout mouse models, we evaluated their contributions to antibody-mediated virus control. Remarkably, depletion of NK cells or genetic deficiency of T-cells (**Supplementary Figures S3B, C**) did not impact the effectiveness of Wen3-mediated virus control, suggesting that both cell types are dispensable for this process. This comprehensive analysis suggests that neutrophils, NK cells, and T-cells are dispensable for Wen3-mediated virus control.

### Patrolling monocytes are crucial for the antiviral activity of nAbs

**Figure 4:**
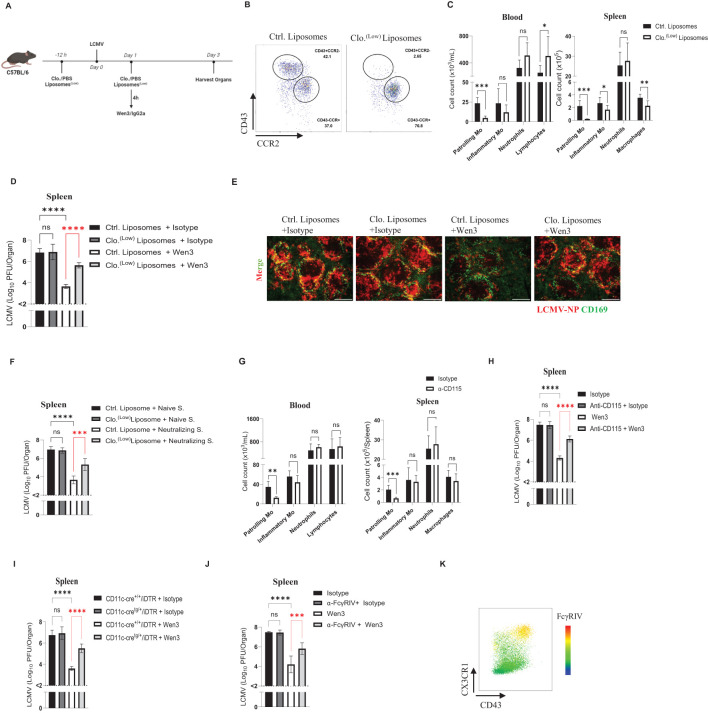
Contribution of patrolling monocytes for antiviral activity of Wen3. **(A)** Schematic presentation of the experiments in **(B-E)**. **(B)** Flow cytometric analysis of monocyte subsets in the peripheral blood and spleen of control or clodronate liposomes-treated mice 3 days post infection (d.p.i.). Animals were treated with 0.5 µL/g control or clodronate liposomes (Clo. ^(low)^) on day -1 and 1. Mice were infected with LCMV-WE (2x10^5^ PFU) on day 0. See (**Supplementary Figure S2**) for gating strategy of the monocyte subsets. **(C)** Numbers of patrolling monocytes (-Mo), inflammatory monocytes (-Mo), neutrophils, and lymphocytes/macrophages in the peripheral blood and spleen of control or clodronate liposome-treated mice 3 d.p.i. Animals were treated with 0.5µL/g control or clodronate liposomes (Clo. ^(low)^) on day -1 and 1. mice were infected with LCMV-WE (2x10^5^ PFU) on day 0. Results show the pooled data from two independent experiments with similar results. (n=3–5 mice/group/experiment). **(D)** Virus titres (3 d.p.i.) in spleen of WT mice infected with LCMV-WE (2x10^5^ PFU) on day 0 and treated on day 1 with Wen3 (350 µg) or isotype (IgG2a), On day -1 and day 1 mice were treated with 0.5µL/g bodyweight control or clodronate liposomes (Clo. ^(low)^). Results show the pooled data from three independent experiments with similar results (n=3–4 mice/group/experiment). **(E)** Spleen sections collected 3 d.p.i. were stained for CD169 (green) and LCMV nucleoprotein (−NP) (red). Shown are the representative merged images from three independent experiments. Scale bar= 300 µm. **(F)** Virus titres (3 d.p.i.) in spleen of WT mice infected with LCMV-WE (2x10^5^ PFU) on day 0 and treated on day 1 with neutralizing serum (Neutralizing S.) or naïve serum (see methods). On day -1 and day 1 mice were treated with 0.5µL/g control or clodronate liposomes (Clo. ^(low)^). Results show the pooled data from two independent experiments with similar results (n=3–5 mice/group/experiment). **(G)** Numbers of patrolling monocytes (-Mo), inflammatory monocytes (-Mo), neutrophils, along with lymphocytes in the peripheral blood or macrophages in spleen (3 d.p.i.) of mice treated with anti-CD115 or Isotype. Mice were infected with LCMV-WE (2x10^5^ PFU) on day 0, preceded by treatment with 100 µg anti-CD115 or isotype (rat IgG2b) on day -1 and again on day 1. Results show the pooled data from two independent experiments with similar results (n=3 mice/group/experiment). **(H)** Shown are the virus titres (3 d.p.i.) in spleen of WT mice infected with LCMV-WE (2x10^5^ PFU) on day 0 and treated on day 1 with Wen3 (350 µg) or isotype (IgG2a). On day -1 and day 1 mice were treated with 100 µg anti-CD115 or isotype. Results show the pooled data from two independent experiments with similar results (n=3 mice/group/experiment). **(I)** Shown are the virus titres (3 d.p.i.) in spleen of CD11c-cre^+/+^/iDTR and CD11c-cre^tg/+^/iDTR mice infected with LCMV-WE (2x10^5^ PFU) on day 0 and treated on day 1 with Wen3 (350 µg) or isotype (IgG2a), on day -1 and day 1 mice were treated with diphtheria toxin 12ng/g bodyweight. Results show the pooled data from two independent experiments with similar results (n=3–4 mice/group/experiment). **(J)** Shown are the virus titres (3 d.p.i.) in spleen of WT mice infected with LCMV-WE (2x10^5^ PFU) on day 0 and treated on day 1 with Wen3 (350 µg) or isotype (IgG2a), mice were treated with 200 µg 9E9 or isotype for blocking FcγRIV. Results show the pooled data from three independent experiments with similar results (n=3–4 mice/group/experiment). **(K)** Heat map showing the expression of FcγRIV on CD11b^+^Ly6G^-^ population gated from CD45^+^Lin^-^(CD45R^+^ CD8^+^CD4^+^), 24 hour following infection with LCMV. Statistical analysis was performed by using the Student’s t-test **(C, G)** or One-way ANOVA test **(D, F, H–J)**. *p < 0.05, **p < 0.01, ***p < 0.001, ****p < 0.001, ns, non-significant. Horizontal dotted lines indicating the detection limit.

Building on our earlier observation of the selective binding of Wen3 to patrolling monocytes, as well as the increased FcγRIV expression on these cells upon infection (**Figures 2A-D**), we aimed to delve deeper into their potential role in antibody-mediated immune responses. To directly assess the significance of patrolling monocytes in IgG-mediated virus control, we employed a targeted depletion approach using a low dose of clodronate, as illustrated in **Figure 4A**. Administration of this clodronate dose effectively resulted in the complete elimination of patrolling monocytes throughout the experiment period (**Figures 4B, C**). Interestingly, depletion of patrolling monocytes resulted in loss of the Fc-dependent virus control of nAbs. (**Figures 4D, E**). To further investigate whether this effect is based on specific features of the Wen3, or it is generalized to other nAbs, we repeated the monocyte depletion procedure as described earlier and treated mice with neutralizing serum. Intriguingly, we observed a reduction in the activity of the neutralizing serum in the absence of patrolling monocytes (**Figure 4F**), thus further supporting the generalization of our findings.

To address concerns regarding potential toxicity induced by clodronate, we opted to deplete monocytes using CD115, a receptor known for its high expression on monocyte subsets (41). Administration of CD115 resulted in more than a 60% reduction in the numbers of patrolling monocytes while leaving inflammatory monocytes unaffected (**Figure 4G**). Consistent with our earlier observations, depletion of patrolling monocytes led to loss of Fc-mediated virus control (**Figure 4H**). Moving forward, we utilized a mouse model (CD11c-cre/iDTR) in which diphtheria toxin receptor (DTR) expression is induced under the CD11c/Itgax promoter (42), allowing for the depletion of dendritic cells as well as patrolling monocytes by diphtheria toxin administration. Consistent with the previously observed role of patrolling monocytes, CD11c-cre/iDTR mice exhibited a loss of the Fc-dependent virus control of nAbs (**Figure 4I**).

Having identified patrolling monocytes as essential for Fc-mediated virus control by nAbs, our next objective was to investigate which activatory FcγR is involved in this process. IgG2a antibodies, such as Wen3, were reported to have a very weak or no affinity for FcγRIII (11). To further investigate which FcγR involved in Wen3-mediated virus control we utilized FcγRIII-KO mice, and as expected, FcγRIII-KO mice did not influence the efficacy of Wen3 (**Supplementary Figure S3D**), demonstrating a comparable reduction in virus titres to WT mice. Having confirmed the dispensability of FcγRIII, we turned our attention to FcγRIV, given its high affinity to IgG2a antibodies like Wen3. Remarkably, blocking of FcγRIV led to loss of Fc-mediated virus control (**Figure 4J**), which aligns well with the observed increase in expression of FcγRIV on patrolling monocytes during infection (**Figure 4K**). These findings collectively confirm the pivotal role of patrolling monocytes in regulating the Fc-dependent virus control of nAbs.

### Patrolling monocytes exhibit direct cytotoxicity against virus-infected cells

**Figure 5:**
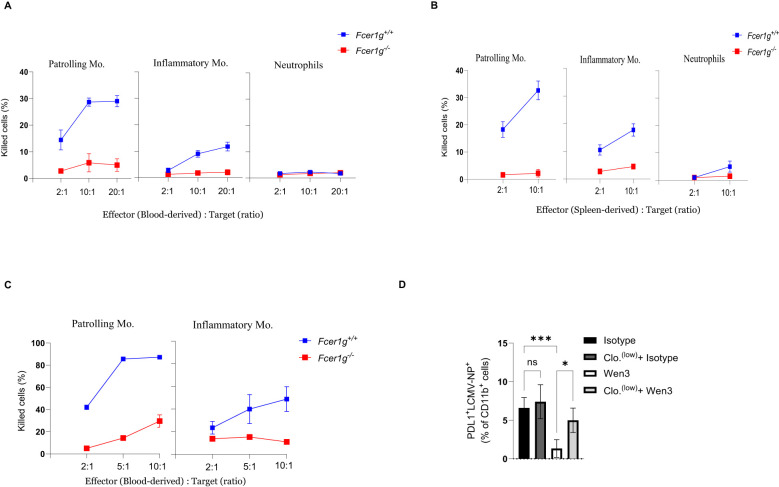
ADCC activity of monocyte subsets against virus infected cells. **(A, B)** Shown is the capacity of blood-derived **(A)** and spleen-derived **(B)** patrolling monocytes, inflammatory monocytes, and neutrophils from Fcer1g^+/+^ (WT) and Fcer1g^-/-^ mice to mediate antibody-dependent killing of infected bone marrow-derived dendritic cells *in vitro* at the indicated effector to target cell ratios in the presence of the Wen3. Shown is one out of three independent experiments, each were done in triplicate. **(C)** Shown is the capacity of blood-derived patrolling monocytes and inflammatory monocytes (Fcer1g^+/+^) and Fcer1g^-/-^ (FcγR-KO) to mediate antibody-dependent killing of B-cells *in vitro* at the indicated effector to target cell ratios in the presence of CD20. Shown is one out of three independent experiments, each was done in triplicate. **(D)** Percentage of PDL1^+^LCMV-NP^+^ cells (gated from CD11b^+^ cells, not shown) from spleen of WT mice infected with LCMV-WE on day 0 and treated on day 1 with Wen3 (350 µg) or isotype (IgG2a), mice were treated with 0.5 µL/g of clodronate liposomes 12 hours before infection and again 24 hours post-infection to deplete patrolling monocytes, as control group mice were treated with PBS. Shown is one out of two independent experiments with similar results. (n=5 mice/group/experiment). Statistical analysis was performed by using the One-way ANOVA test **(D)**. *p < 0.05, ***p < 0.001, ns, non-significant.

To further dissect the mechanism underlying patrolling monocyte-mediated viral control, we investigated whether direct elimination of infected cells by patrolling monocytes contributes to this phenomenon. We employed an *in vitro* co-culture system consisting of patrolling monocytes and LCMV-infected target cells to assess the cytolytic activity of patrolling monocytes against virus-infected cells. We utilized bone marrow-derived dendritic cells (BMDCs) from FcγR-KO mice as target cells and infected them *in vitro* for 48 hours to allow for viral propagation and antigen presentation. After 48 hours, most of the infected BMDCs were found to express the epitope for Wen3 (**Supplementary Figure S4A**). As effector cells, we utilized patrolling monocytes, inflammatory monocytes, and neutrophils. Interestingly and in line with our earlier observations, among all tested effector cells, both blood-derived and spleen-derived patrolling monocytes exhibited the highest killing capacity against virus-infected cells (**Figures 5A, B**). To further investigate whether the killing capacity of patrolling monocytes is specific to virus-infected cells or represents a broader activity, we utilized naïve B-cells as target cells. Interestingly, patrolling monocytes exhibited a significant ability to mediate killing of B-cells using CD20 antibody *in vitro* compared to inflammatory monocytes (**Figure 5C**). Indeed, our *in vivo* data further supported this observation, revealing a reduction in the number of LCMV-infected cells expressing high levels of PD-L1 following Wen3 treatment (**Figure 5D**). This decrease is likely attributed to their elimination through ADCC mechanisms.

To address whether FcγR-mediated killing could also be mediated by non-neutralizing antibodies, we tested the KL53 clone, a non-neutralizing IgG2a antibody directed against the LCMV nucleoprotein (NP). *In vitro*, KL53 exhibited only minimal killing capacity when co-cultured with patrolling monocytes, substantially lower than that observed with Wen3 (**Supplementary Figure S5A**). These findings suggest that not all virus-specific antibodies are equally capable of triggering FcγR-mediated effector functions. However, given that KL53 targets NP rather than the surface-expressed glycoprotein (GP), differences in antigen accessibility must be considered when interpreting these results. Future studies will be required to more directly address the role of non-neutralizing GP-targeting antibodies in FcγR-dependent viral clearance.

These compelling results strongly suggest that the interaction between activatory FcγRs on patrolling monocytes and virus-specific antibodies, plays a pivotal role in controlling virus infection by eliminating virus-infected cells.

### Numbers of patrolling monocytes dictate passive immunization outcomes

**Figure 6:**
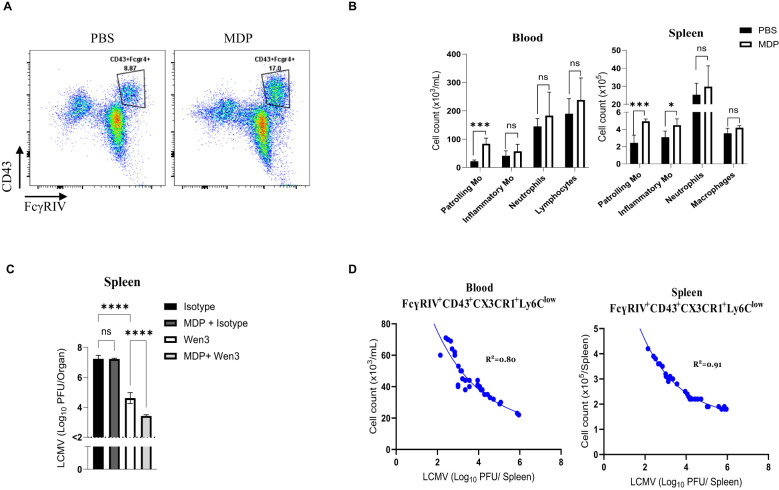
Numbers of patrolling monocytes dictate passive immunization outcomes **(A)** Flow cytometry plots depict the absolute numbers of patrolling monocytes in the peripheral blood of mice infected with LCMV-WE (2x10^5^ PFU) and treated twice, on day -1 and day 1 of infection, with MDP (muramyl dipeptide) or PBS as a control. **(B)** Numbers of patrolling monocytes (-Mo), inflammatory monocytes (-Mo), neutrophils, and lymphocytes/macrophages 3 days post infection (d.p.i.) in the peripheral blood and spleen of MDP or PBS treated mice. Mice were treated with MDP or PBS on day -1 and 1. Mice were infected with LCMV-WE (2x10^5^ PFU) on day 0. Results show the pooled data from two independent experiments with similar results (n=4–5 mice/group/experiment). **(C)** Shown are the virus titres 3 d.p.i. in the spleen of WT mice infected with LCMV-WE (2x10^5^ PFU) on day 0 and subsequently treated on day 1 with either Wen3 (350 µg) or an isotype control (IgG2a). Mice were treated twice with either MDP or PBS on day -1 and day 1. Results show the pooled data from two independent experiments with similar results (n=4–5 mice/group/experiment). **(D)** Shown are the cell numbers of patrolling monocytes in blood (left) and spleen (right) plotted against the LCMV titres (3 d.p.i.) of WT mice infected with LCMV-WE on day 0 and treated on day 1 with Wen3 (350 µg). Statistical analysis was performed by using the Student’s t-test **(B)** or One-way ANOVA test **(C)**. *p < 0.05, ***p < 0.001, ****p < 0.001, ns, non-significant. Horizontal dotted lines indicating the detection limit.

Next, we aimed to investigate how the levels of patrolling monocytes impact passive immunization effectiveness. We took advantage of the previously described role of muramyl dipeptide (MDP) in increasing numbers of patrolling monocytes through converting inflammatory monocytes into patrolling monocytes by triggering the NOD2 receptor (21). As expected, the treatment with MDP leads to an increase in numbers of patrolling monocytes (**Figures 6A, B**). In line with our previous observations, administering Wen3 to mice treated with MDP led to a substantial improvement in spleen virus control (**Figure 6C**). This highlights the pivotal role of patrolling monocytes in shaping passive immunization efficacy. Moreover, upon reviewing all previously conducted experiments, we found a direct correlation between Wen3-mediated virus control and patrolling monocyte counts in blood and spleen (**Figure 6D**). Overall, our findings emphasize the critical influence of patrolling monocyte frequencies on passive immunization outcomes. They shed light on the intricate relationship between patrolling monocytes and antibody-driven immune responses, highlighting their potential as key factors in antiviral defense strategies.

## Discussion

The results presented here provide a novel mechanism underlying FcγR-mediated virus control and highlight the crucial role of patrolling monocytes in shaping antiviral immunity of nAbs. Our findings demonstrate that activatory FcγRs play a pivotal role in facilitating the early control of viral titres by virus-specific nAbs. This is evidenced by the significant reduction in virus levels following treatment with both broad nAbs and the monoclonal antibody Wen3 in WT mice compared to FcγR deficient mice. Importantly, despite similar neutralizing capacities of serum from both WT and KO mice, the presence of activatory FcγRs in WT mice confers enhanced virus control beyond neutralization in circulation. Our study reveals a dependency on activatory FcγRs for virus control via nAbs, particularly evident in the spleen but not in the liver. This observation aligns with previous findings suggesting distinct organ-specific Fc-effector pathways responsible for target cell depletion (7).

The role of FcγRs in mediating IgG effector functions has been extensively studied, revealing their crucial involvement in various immune processes. However, the specific role of FcγRs on different effector cells in mediating IgG effector functions during virus infection is less well characterized (18). This knowledge gap arises from the inherent challenge of studying effector functions in the context of viral infections (30). Unlike autoimmune diseases, where the immune response is more localized, viral infections trigger complex immune responses involving multiple cell types recruited to sites of inflammation. This complexity makes it challenging to pinpoint which specific cell types are crucial for antibody-dependent effects (20).

In line with earlier observations, our findings highlight the essential role of FcγR-mediated mechanisms in virus control mediated by broad nAbs (37). *In vivo*, nAbs exert their antiviral effects through various mechanisms, which include both neutralization and antibody effector functions. Neutralization is typically defined by *in vitro* assays wherein nAbs block viral entry into target cells (30, 43, 44). In an ideal scenario, *in vitro* neutralization assays should accurately replicate the *in vivo* conditions where nAbs interact with viruses. However, these assays frequently employ conditions that deviate significantly from the *in vivo* environment. Thus, interpreting *in vivo* outcomes in the context of these *in vitro* assays might not be optimal, since the relative contributions of different antiviral mechanisms to the *in vivo* efficacy of antibodies are missing *in vitro (* 7).

Our results affirm previous reports on the involvement of myeloid cells, particularly patrolling monocytes, in ADCC activity in mice (10, 20). This aligns with the observed role of patrolling monocytes in mediating killing of virus infected cells *in vitro*. Additionally, the pivotal role of patrolling monocytes in antibody-mediated virus control was further elucidated through multiple lines of evidence. Depletion of patrolling monocytes led to the loss of Fc-dependent virus control of nAbs, highlighting the essential role of these cells in this process. Notably, selective depletion of inflammatory monocytes or macrophages did not impair Wen3-mediated virus control, underscoring the unique contribution of patrolling monocytes in this context.

Moreover, our investigations highlight the kinetics of patrolling monocyte levels as a critical determinant of passive immunization efficacy. Augmenting patrolling monocyte levels through interventions like MDP administration enhances virus control, suggesting novel avenues for passive immunization strategies. These findings have implications for the development of novel therapeutic strategies aimed at bolstering antiviral immunity and mitigating the pathogenic consequences of chronic viral infections.

Overall, our study provides comprehensive insights into the mechanisms of nAbs-mediated virus control via FcγRs and highlights the indispensable role of patrolling monocytes in this process. These findings have important implications for the development of novel antiviral vaccines and underscore the potential therapeutic value of nAbs in combating viral infections. Further research into the specific effector mechanisms involved in antibody-mediated virus control, as well as the interplay between patrolling monocytes and other immune cells, is warranted to fully harness the potential of this immune response in clinical settings.

### Study limitations

Although we demonstrated the direct involvement of patrolling monocytes in killing virus-infected cells via ADCC mechanisms, we did not specifically investigate the possibility of patrolling monocytes phagocytizing opsonized viruses. Considering the well-documented high phagocytic capability of patrolling monocytes, it is highly plausible that they may also engage in the phagocytosis of opsonized viruses, further contributing to the elimination of viral infections. Moreover, patrolling monocytes are known for their non-classical effector functions, including, trogocytosis, and interactions with tissue-resident immune cells that may shape the broader antiviral response. Although they are generally thought to lack robust expression of canonical cytotoxic molecules such as perforin and granzyme B, this does not exclude their participation in non-classical ADCC-like activity. Further investigations are warranted to explore this aspect comprehensively.

## Experimental procedures

### Mice

This study utilized mice sourced from various strains, including C57BL/6 (wild type, WT) obtained from Jackson Laboratory, Fcer1g-KO (FcγR-KO or *Fcer1g^-/-^*) mice, siblings served as controls, CD11c-cre and iDTR mice were provided by Prof. Dr. Ari Waisman, Johannes-Gutenberg-University Mainz, to WH, FcγRIII-KO were acquired from Prof. Dr. Falk Nimmerjahn, Friedrich-Alexander-University Erlangen, and Tcrb-KO (Tcrb^-/-^) mice were acquired from Jackson Laboratory. Mice, maintained on the C57BL/6 genetic background (when needed back-crossed at least 10 times) and bred as homozygotes were utilized. Male and female mice aged between 8 to 16 weeks were selected for experimentation. To ensure unbiased allocation, age and sex-matched animals were randomly assigned to different treatment groups. All mice were maintained in single ventilated cages under specific-pathogen-free conditions. Animal experimentation was conducted under the authorization of the Nordrhein Westfalen Landesamt für Natur, Umwelt und Verbraucherschutz (Recklinghausen, Germany), and adhered strictly to the German law for animal protection and institutional guidelines at the Ontario Cancer Institute of the University Health Network and at McGill University.

### *In vivo* virus infection

Mice were intravenously injected with LCMV-WE at doses of 2x10^5^ PFU, unless otherwise stated. Wen3 was administered intravenously 15–18 hours following infection unless otherwise indicated. The LCMV strain WE was originally obtained from Prof. Rolf Zinkernagel (Institute of Experimental Immunology, ETH, Zurich, Switzerland). The viral propagation process involved culturing on BHK-21 cells, which were purchased from ATCC (CRL-8544), at a multiplicity of infection (MOI) of 0.01. Following, the concentration of LCMV was quantified using the methodology described below (Fink et al., 2006).

### Plaque assay

Mice were intravenously infected with LCMV at the specified dosages. Virus titres were assessed using a plaque-forming assay. Evaluation of LCMV titres involved smashing of organs in Dulbecco’s modified Eagle medium (DMEM) supplemented with 2% fetal calf serum (FCS). Serial 1:3 dilutions of the samples were prepared, with alternate dilutions transferred onto preseeded confluent MC57 fibrosarcoma cells. Following a 2h incubation at 37°C, an overlay was applied, and the viral preparation was re-incubated at 37°C. Plaques were counted 48h later via LCMV NP staining. Cells were fixed with a 4% formaldehyde solution, permeabilized using a 1% Triton-X solution and subsequently blocked with 10% FCS in phosphate-buffered saline. Blocked cells were stained with anti-LCMV NP (VL4) antibody (made in house), followed by incubation with an ECL-conjugated anti-rabbit-IgG secondary antibody. Plaques were visualized utilizing a color reaction employing a solution composed of 0.2 M Na_2_HPO_4_, 0.1 M citric acid, 30% H_2_O_2_ and *o*-phenylenediamine dihydrochloride, with all chemicals obtained from Sigma-Aldrich.

### LCMV-neutralizing antibodies

LCMV immune serum (Neutralizing serum) was collected from C57BL/6 mice 90 or 120 days after infection with 2*10^5^ PFU LCMV-WE, mice were re-boosted with 2*10^5^ PFU LCMV-WE 15 days before serum collection. Serum samples were pooled from 20–30 mice and subsequently tested for LCMV titres and virus neutralizing activity using a focus-forming assay. A hybridoma cell line producing Wen3 or KL53 was originally obtained from Prof. Rolf Zinkernagel (Institute of Experimental Immunology, ETH, Zurich, Switzerland). Antibodies (Wen3 and KL53) were purified from hybridoma supernatants by affinity chromatography using Protein G Sepharose.

### LCMV neutralization Assay

For measuring neutralizing activity of Wen3 or serum from immunized mice, serum was prediluted (1:40) followed by complement system inactivation at 56°C for 30 min. For analysis of IgG kinetics, diluted samples were treated with 2-mercaptoethanol (0.1 M) for removal of IgM. Wen3 was utilized at an initial concentration of 1 mg/ml. Sera/Wen3 was titrated 1:2 over 12 steps and was incubated with 60 PFU of LCMV. After a 90-min incubation at 37°C, MC57 mouse fibroblast cells were added to each well and evenly distributed on a plate shaker. After cell adhesion (typically 2 to 3 hours later), 1% methylcellulose was added, and the cells were further incubated for 48 hours. Upon confluence, the cells were fixed with 4% formaldehyde in PBS and permeabilized with 1% Triton X-100. Foci were visualized by staining for the NP using the VL-4 antibody. Titres were determined based on half-maximal inhibition values. Antibody titres are presented as twofold dilution steps (−log_2_) times the predilution (that is, × 40).

### Bone marrow chimeras

For bone marrow chimera experiments, female Fcer1g-KO mice aged 10–12 weeks underwent irradiation with a dose of 9.5 Gy. The following day, bone marrow was aseptically isolated from donor mice and intravenously administered to the irradiated recipients. On day 10 post-irradiation, mice received treatment with clodronate liposomes to deplete tissue resident macrophages. Following a 50-day reconstitution period, the mice were considered suitable to be used in experimental procedures.

### Histology

Histological analysis of snap-frozen spleen tissues involved staining with various monoclonal antibodies targeting LCMV-NP (Clone: VL4; made in-house, 1:1), Biotin-SP anti-rat (112-065-167, Jackson Immuno Research, 1:150), Streptavidin-APC (405207, Biolegend, 1:75), anti-F4/80 (53-4801-82, Thermo Fisher Scientific, 1:100) and anti-CD169 (130-124-896, Mylteni Biotec, 1:100).

In summary, the organs were embedded in tissue-tek and promptly snap-frozen in liquid nitrogen. The frozen tissue was then sliced into 8.0 µm thick sections. Following sectioning, the slides were air-dried and fixed in acetone for 10 min. Next, the slides were placed in a staining dish and incubated with blocking buffer (PBS with 4% FBS) for 25 min at room temperature to prevent nonspecific binding. All antibody mixtures were diluted in PBS with 2% FBS and incubated for 45 min at room temperature. Between the different staining steps, the slides were washed three times in PBS. Initially, the slides were stained with the LCMV-NP antibody. Subsequently, the Biotin-SP anti-rat antibody was used to localize VL-4 binding. Lastly, the remaining antibodies were mixed at the specified ratios above and applied to the slides. Following washing, one drop of fluorescence mounting media was added to each slide, followed by a coverslip. The slides were then left to dry in darkness for at least 30 min before image acquisition. Images were captured using the Keyence BZ-9000 (Keyence, Osaka, Japan).

### Cell depletion and blocking

Administration of antibodies was conducted intraperitoneally (i.p.) one day prior to infection and one day post-infection, unless stated otherwise. Depletion antibodies utilized were CD115 (Clone: AFS98), Ly6G (Clone: 1A8), NK (Clone: PK136), all procured from BioXcell, and each was administered at a dosage of 200 µg per mouse (except CD115 100 µg). Additionally, CCR2 (Clone: MC21) was obtained from Prof. Mattias Mack and administered at a dosage of 25 µg per mouse. For the blockage of FcγRIV, anti-FcγRIV (Clone: 9E9) was administered intraperitoneally at a dosage of 200 µg per mouse. Monocyte depletion was achieved by administering 0.5 µL/g mice weight (10 µL/20 g mice) of clodronate liposomes one day prior to infection and 4 hours before Wen3 treatment, unless otherwise specified. Macrophage depletion involved the administration of 200 µL clodronate intraperitoneal 24-30h prior to infection.

### Generation of bone-marrow derived dendritic cells

Bone marrow was harvested from the femurs and tibias of Fcer1g-KO mice. Red blood cells were lysed using RBC lysis buffer (PAN Biotech, cat#P10-90100), and the remaining cells were collected following centrifugation. These bone marrow cells were then resuspended at a concentration of 1.5 × 10 (6) cells/mL in complete RPMI containing 20 ng/ml of GM-CSF and 10 ng/ml of IL4 (Peprotech) and incubated at 37°C with 5% CO2 for 6 days. On day 2 of incubation, 75 ng/ml of GM-CSF and 30 ng/ml of IL4 were added in half the amount of the original media. After 6 days, non-adherent cells were collected and washed, then infected with LCMV-WE at an MOI of 0.1. 48 hours post-infection, the cells were harvested and labeled with CFSE before being co-cultured with effector cells.

### *In-vitro* ADCC assay

Naive neutrophils, patrolling and inflammatory monocytes were isolated from blood and spleen of Fcer1g-WT and Fcer1g-KO mice using flow cytometric sorting on a FACS Aria III (BD) after enrichment by positive selection of B cells (Cat#130-049-501) and T cells (Cat#130-094-973) using Miltenyi beads. These effector cells were then combined at specified effector to target cell ratios in 96-well plates with Wen3 antibody at a concentration of 4 µg/mL. As a target cell, infected bone marrow-derived dendritic cells from Fcer1g-KO mice was employed as described above, for some experiments naïve B cells positively selected by B220 (Cat#130-049-501) were employed as target cells with CD20 antibody in concentration of 1 µg/mL. Following incubation for 16–20 hours at 37°C, total cell numbers were determined, and flow cytometric analysis was performed to quantify the different cell types present in the culture.

### Cytokine measurement

Serum cytokines were analyzed with LEGENDplex™ (BioLegend, San Diego, USA) according to the instructions and recommendation of the manufacturer.

### Statistical analysis

Data is expressed as mean± SD. Student’s *t*-test and one-way ANOVA was used to detect statistically significant differences between groups unless otherwise mentioned. The level of statistical significance was set at *P*<0.05.

## Data Availability

The original contributions presented in the study are included in the article/**Supplementary Material**. Further inquiries can be directed to the corresponding authors.

## Supplementary material

Supplementary Figure 1 Transferred virus-specific antibodies predominantly act through activatory fc receptors. **(A)** *In vitro* focus forming assay showing the neutralizing capacity of the serum collected from immune WT mice and Wen3. Pooled sera were collected from mice infected for 90 days with LCMV-WE. Shown is the pooled data from two independent experiments, each were done in triplicate. **(B)** Levels of IFN-γ and IFN-*α* in the serum were measured by LEGENDplex™ assay 3 d.p.i. Fcer1g^+/+^ and Fcer1g^-/-^ mice infected with LCMV-WE (2x10^5^ PFU) on day 0 and treated on day 1 with Wen3 (350 µg) or isotype (IgG2a). Results show the pooled data from three independent experiments with similar results (n=3–4 mice/group/experiment). (C and D) Shown are the virus titres (3 d.p.i.) in spleen of WT (Fcer1g^+/+^) and FcγR-KO (Fcer1g^-/-^) mice infected with LCMV-WE (2x10^5^ PFU) on day 0 and treated 12 hours before infection with **(C)** Neutralizing serum/naïve serum, **(D)** Wen3(350 μg) or isotype (IgG2a). Results show the pooled data from two independent experiments with similar results (n=3–4 mice/group/experiment). Statistical analysis was performed by using the One-way ANOVA test **(B-D)**. *p < 0.05, **p < 0.01, ***p < 0.001, ****p < 0.001. Horizontal dotted lines indicating the detection limit.

Supplementary Figure 2 Gating strategy for monocyte subsets. **(A, B)** Flow cytometric analysis of cell populations in the peripheral blood and spleen, patrolling and inflammatory monocytes were identified based on the expression of CD43 and CX3CR1 after excluding Lineage positive (CD45R+CD4+CD8++NK+), Ly6G for blood **(A)**, in addition to F4/80 for spleen **(B)**. The histograms depict the expression of Ly6C, CCR2, CD64 (FcγRI), and CD16.2 (FcγRIV) on inflammatory monocytes (marked in red) and patrolling monocytes (marked in blue).

Supplementary Figure 3 Neutrophils, NK cells, and T-cells, as well as FcγR3 role in mediating Wen3-driven virus control. **(A, B)** Shown are the virus titres 3 d.p.i. in spleen of WT mice infected with LCMV-WE on day 0 and treated on day 1 with Wen3 (350 µg) or isotype (IgG2a). On day -1 and day 1 mice were treated with depleting antibody **(A)** Ly6G (clone: 1A8) or isotype (rat IgG2a, κ), or **(B)** NK depleting antibody (clone: PK136) or isotype (mouse IgG2a, κ). Shown is one out of three independent experiments with similar results (n=4–5 mice/group/experiment). **(C)** Virus titres (3 d.p.i.) in spleen of WT and Tcrb^-/-^ mice infected with LCMV-WE on day 0 and treated on day 1 with Wen3 (350 µg) or isotype (IgG2a). Shown is one out of three independent experiments with similar results (n=4–5 mice/group/experiment). **(D)** Shown are the virus titres (3 d.p.i.) in spleen of WT and FcgR3^-/-^ mice infected with LCMV-WE on day 0 and treated on day 1 with Wen3 (350 µg) or isotype (IgG2a). Shown is one out of three independent experiments with similar results (n=4–5 mice/group/experiment). Statistical analysis was performed by using the One-way ANOVA test **(A-D)**. *p < 0.05, **p < 0.01, ***p < 0.001, ****p < 0.001. Horizontal dotted lines indicating the detection limit.

Supplementary Figure 4 Bone marrow-derived dendritic cells express the LCMV-GP on its surface. **(A)** Histogram overlays show Wen3 staining in naïve (uninfected, red) and LCMV-WE–infected (blue) bone marrow-derived dendritic cells (BMDCs). BMDCs from Fcer1g^-/-^ (FcγR-KO) mice were infected *in vitro* with LCMV-WE at a multiplicity of infection (MOI) of 1 and incubated for 48 hours to allow for viral propagation and antigen presentation. Wen3 epitope expression was assessed by flow cytometry. Representative of at least two independent experiments performed in triplicate.

Supplementary Figure 5 Non-neutralizing antibodies exhibit reduced capacity to exert Fc receptor–mediated elimination of virus-infected cells *in vitro* **(A)** Shown is the capacity of blood-derived patrolling monocytes from Fcer1g^+/+^ (WT) and Fcer1g^-/-^ mice to mediate antibody-dependent killing of infected bone marrow–derived dendritic cells *in vitro* at the indicated effector-to-target cell ratios, in the presence of Wen3 or KL53 antibodies. The experiment was performed once with four technical replicates.
