# Supplementary material for: Patrolling monocytes mediate virus neutralizing IgG effector functions: beyond neutralization capacity
Source: Front Immunol. 2025 May 29;16:1600056. doi: 10.3389/fimmu.2025.1600056 (PMC12158932; doi:10.3389/fimmu.2025.1600056)

Supplementary Fig. 1 Transferred virus-specific antibodies predominantly act through activatory Fc receptors

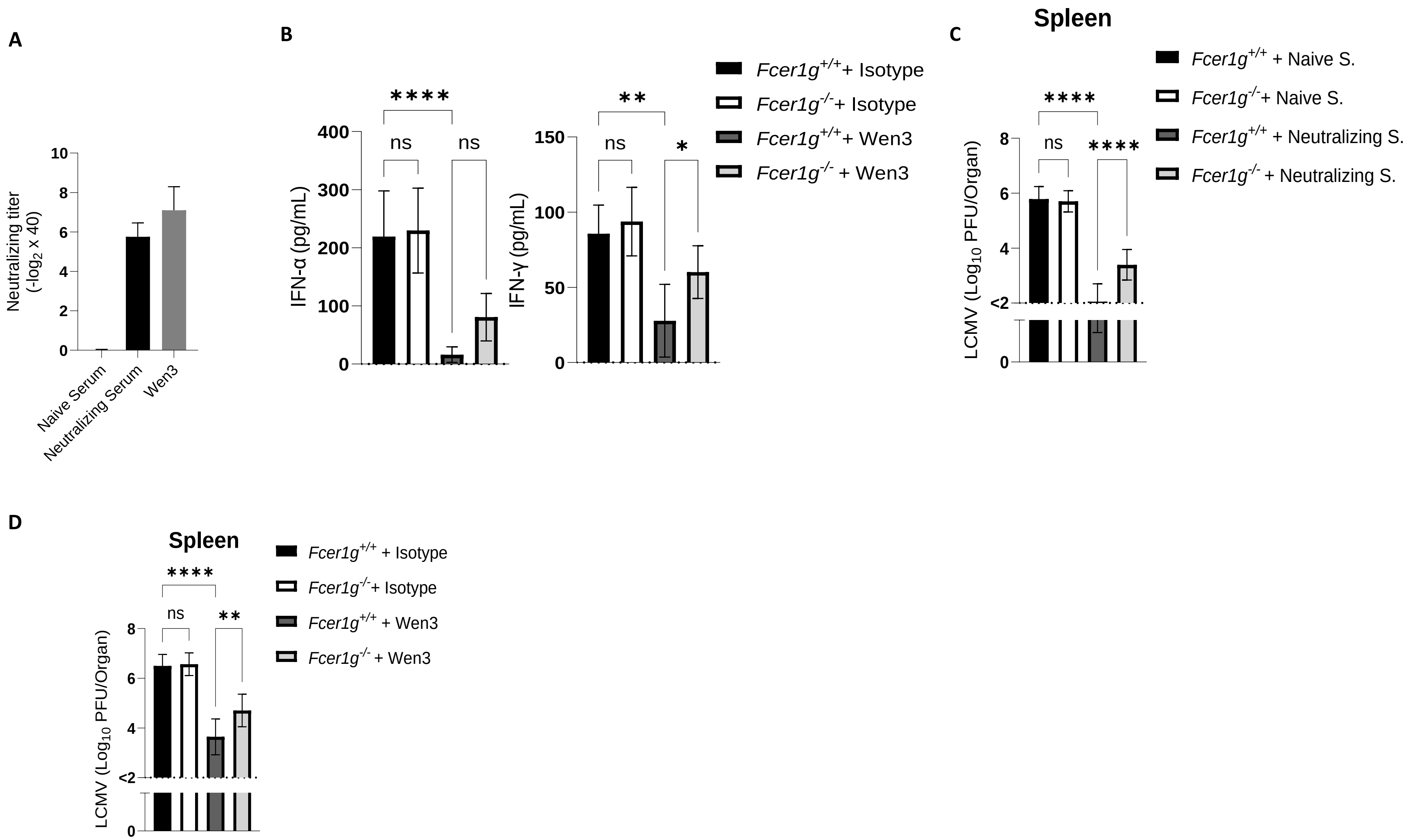

Supplementary Fig. 2: Gating strategy for monocytes

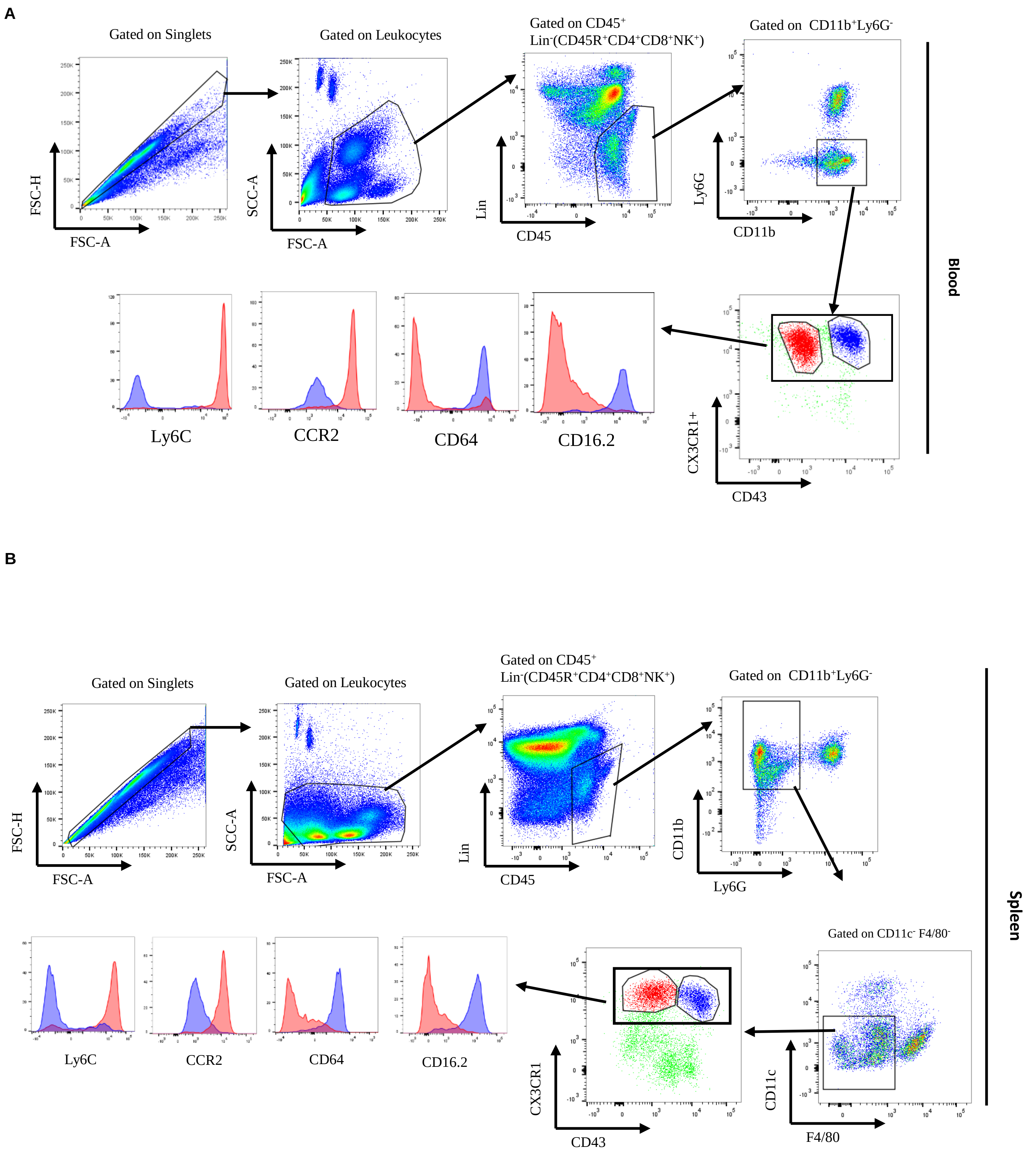

Supplementary Fig.3. Neutrophils, NK cells and T-cells are dispensable for nAbs-mediate virus control

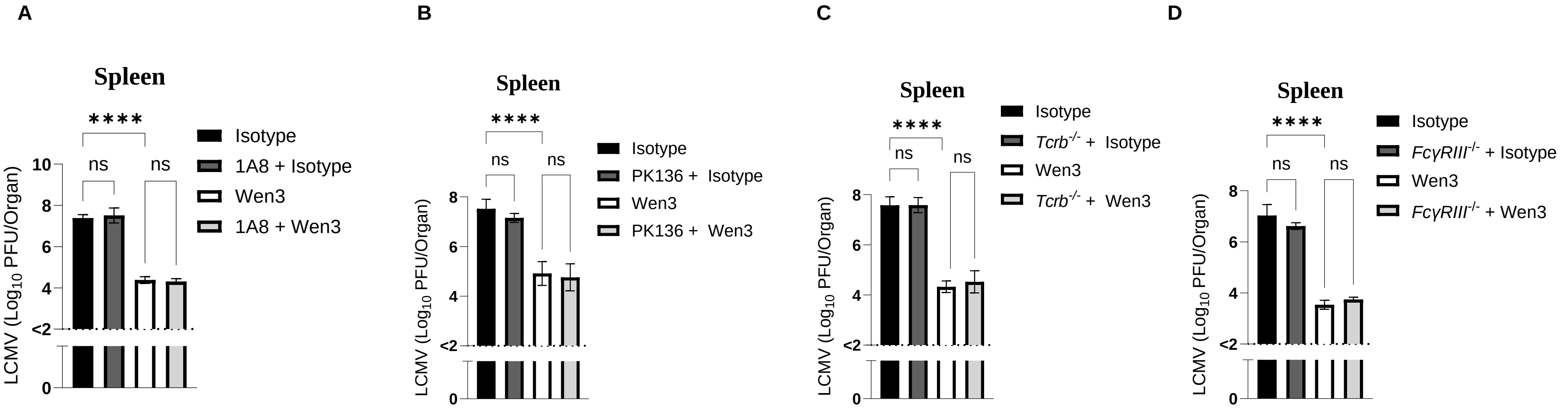

Supplementary Fig.4. Bone marrow-derived dendritic cells express the LCMV-GP on its surface

A

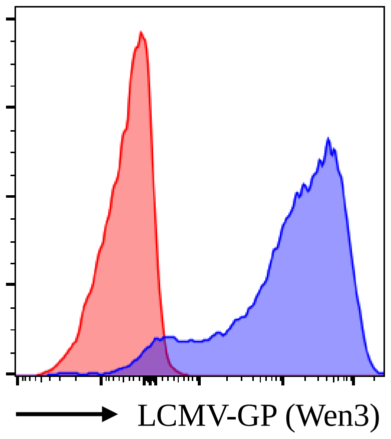

Supplementary Fig.5. Non-neutralizing antibodies exhibit reduced capacity to exert Fc receptor–mediated elimination of virus-infected cells in vitro

A

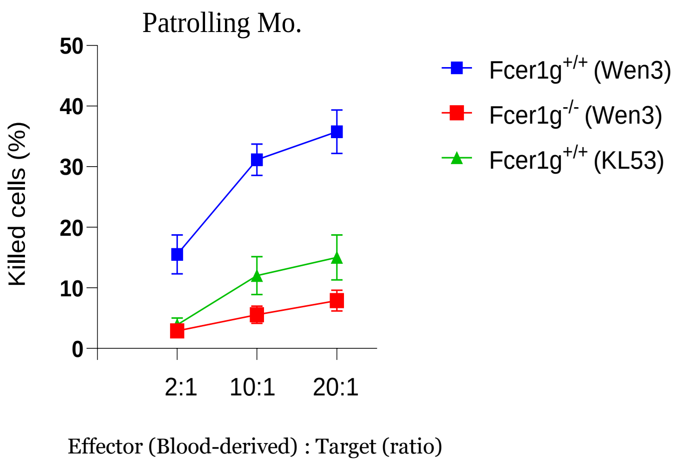

Supplement: Supplementary Figure 1 — Transferred virus-specific antibodies predominantly act through activatory fc receptors. (A) In vitro focus forming assay showing the neutralizing capacity of the serum collected from immune WT mice and Wen3. Pooled sera were collected from mice infected for 90 days with LCMV-WE. Shown is the pooled data from two independent experiments, each were done in triplicate. (B) Levels of IFN-γ and IFN-α in the serum were measured by LEGENDplex™ assay 3 d.p.i. Fcer1g+/+ and Fcer1g-/- mice infected with LCMV-WE (2x10^5^ PFU) on day 0 and treated on day 1 with Wen3 (350 µg) or isotype (IgG2a). Results show the pooled data from three independent experiments with similar results (n=3–4 mice/group/experiment). (C and D) Shown are the virus titres (3 d.p.i.) in spleen of WT (Fcer1g+/+) and FcγR-KO (Fcer1g-/-) mice infected with LCMV-WE (2x10^5^ PFU) on day 0 and treated 12 hours before infection with (C) Neutralizing serum/naïve serum, (D) Wen3(350 μg) or isotype (IgG2a). Results show the pooled data from two independent experiments with similar results (n=3–4 mice/group/experiment). Statistical analysis was performed by using the One-way ANOVA test (B-D). *p < 0.05, **p < 0.01, ***p < 0.001, ****p < 0.001. Horizontal dotted lines indicating the detection limit. [file DataSheet1.pdf]
